# Associations between Ileal Juice Bile Acids and Colorectal Advanced Adenoma

**DOI:** 10.3390/nu15132930

**Published:** 2023-06-28

**Authors:** Hung N. Luu, Chi Thi-Du Tran, Renwei Wang, Mai Vu-Tuyet Nguyen, Mo Thi Tran, Thuy Thi-Van Tuong, Quang Hong Tran, Linh Cu Le, Huong Thi-Thu Pham, Hien Huy Vu, Nam Chi Bui, Hien Thi-Thu Ha, Dung Tuan Trinh, Claire E. Thomas, Jennifer Adams-Haduch, Liudmilla Velikokhatnaya, Robert E. Schoen, Guoxiang Xie, Wei Jia, Paolo Boffetta, Jose C. Clemente, Jian-Min Yuan

**Affiliations:** 1 UPMC Hillman Cancer Center, University of Pittsburgh, Pittsburgh, PA 15232, USA; wangr2@upmc.edu (R.W.); thomas2@fredhutch.org (C.E.T.); adamshaduchj@upmc.edu (J.A.-H.); velilx@upmc.edu (L.V.); yuanj@upmc.edu (J.-M.Y.); 2 Department of Epidemiology, School of Public Health, University of Pittsburgh, Pittsburgh, PA 15261, USA; rschoen@pitt.edu; 3 Vietnam Colorectal Cancer and Polyps Research, Vinmec Healthcare System, Hanoi 10000, Vietnam; v.chittd@vinmec.com (C.T.-D.T.); nvtuyetmai@gmail.com (M.V.-T.N.); ttpmo@yahoo.co.uk (M.T.T.); tgvanthuy@yahoo.com.vn (T.T.-V.T.); tranhongquang@me.com (Q.H.T.); 4 College of Health Sciences, VinUniversity (VinUni), Hanoi 10000, Vietnam; linh.lc@vinuni.edu.vn; 5 Center of Applied Sciences, Regenerative Medicine and Advanced Technologies, Vinmec Healthcare System, Hanoi 10000, Vietnam; 6 Department of Gastroenterology, Vinmec Healthcare System, Hanoi 10000, Vietnam; v.huongptt1@vinmec.com (H.T.-T.P.); drhuyhien.ns@gmail.com (H.H.V.); v.nambc@vinmec.com (N.C.B.); 7 Department of Cytopathology, Vinmec Healthcare System, Hanoi 10000, Vietnam; v.hienhtt@vinmec.com (H.T.-T.H.); trinhtuandung108@gmail.com (D.T.T.); 8 Department of Cytopathology, Tam Anh General Hospital, Hanoi 10000, Vietnam; 9 Division of Gastroenterology, Hepatology, and Nutrition, Department of Medicine, University of Pittsburgh Medical Center, Pittsburgh, PA 15213, USA; 10 University of Hawai’i Cancer Center, University of Hawaii, Honolulu, HI 96813, USA; gxie@cc.hawaii.edu (G.X.); weijia1@hkbu.edu.hk (W.J.); 11 School of Chinese Medicine, Hong Kong Baptist University, Kowloon, Hong Kong; 12 Stony Brook Cancer Center, Stony Brook University, Stony Brook, NY 11794, USA; paolo.boffetta@stonybrookmedicine.edu; 13 Department of Medical and Surgical Sciences, University of Bologna, 40138 Bologna, Italy; 14 Icahn Institute for Genomics & Multiscale Biology, Department of Genetics and Genomic Sciences, Icahn School of Medicine at Mount Sinai, New York, NY 10029, USA; jose.clemente@mssm.edu; 15 Precision Immunology Institute, Icahn School of Medicine at Mount Sinai, New York, NY 10029, USA

**Keywords:** ileal juice, bile acids, biomarker, colorectal adenomas, colorectal cancer

## Abstract

Background: There is an urgent need to identify biomarkers for advanced adenoma, an important precursor of colorectal cancer (CRC). We aimed to determine alterations in ileal juice bile acids associated with colorectal advanced adenoma. Methods: We quantified a comprehensive panel of primary and secondary bile acids and their conjugates using an ultraperformance liquid chromatography triple-quadrupole mass spectrometric assay in ileal juice collected at colonoscopy from 46 study subjects (i.e., 14 biopsy-confirmed advanced adenomas and 32 controls free of adenoma or cancer). Using analysis of covariance (ANCOVA), we examined the differences in bile acid concentrations by disease status, adjusting for age, sex, body mass index, smoking status and type 2 diabetes. Results: The concentrations of hyodeoxycholic acid (HCA) species in ileal juice of the advanced adenoma patients (geometric mean = 4501.9 nM) were significantly higher than those of controls (geometric mean = 1292.3 nM, *p* = 0.001). The relative abundance of ursodeoxycholic acid (UDCA) in total bile acids was significantly reduced in cases than controls (0.73% in cases vs. 1.33% in controls; *p* = 0.046). No significant difference between cases and controls was observed for concentrations of total or specific primary bile acids (i.e., cholic acid (CA), chenodeoxycholic acid (CDCA) and their glycine- and taurine-conjugates) and total and specific major secondary bile acids (i.e., deoxycholic acid and lithocholic acid). Conclusions: Colorectal advanced adenoma was associated with altered bile acids in ileal juice. The HCA species may promote the development of colorectal advanced adenoma, whereas gut microbiota responsible for the conversion of CDCA to UDCA may protect against it. Our findings have important implications for the use of bile acids as biomarkers in early detection of colorectal cancer.

## 1. Introduction

Colorectal cancer (CRC) is the second- and third-most common cancer among men and women worldwide, respectively (estimated age-standardized incidence rates per 100,000: 20.6 in men and 14.3 in women) [1], with approximately 1,360,000 new cases and 690,000 deaths per year [2]. Colorectal cancer is also ranked among the top 20 causes of death globally, and its burden is expected to rise in the coming decades [3]. Adenomatous polyps (or adenomas) are considered the most significant precursor lesion of CRC [4,5]. Patients with a history of an advanced adenoma have approximately a three-fold increased risk of CRC compared to those without adenomas [6]. In the US, the prevalence of any adenoma is approximately 25% among 50 years or older and increased to 50% by age 70 or older [7], whereas advanced adenomas are observed in about 7–10% of subjects undergoing screening. One of the major concerns is the increasing trend of CRC among those who are younger than 50 years of age, in which a 1.5% increase in incidence and an 13% increase in mortality were observed from 2000 to 2013–2014 [8]. Although the reasons for the increasing rate of CRC among young population are unclear, obesity and Western diet (i.e., high saturated fat, high meat and low fiber) are thought to play an important role. To reduce the burden of CRC, there is an urgent need to identify biomarkers associated with advanced adenomas, precursor lesions with high potential to progress to cancer. Biomarkers which are associated with the underlying biological mechanisms for disease progression are preferred since they have potential to elucidate targets for primary prevention of CRC. 

In recent years, impaired bile acid metabolism has been shown to potentially contribute to the pathophysiology of metabolic diseases. Physiologically, bile acids play important roles in metabolic homeostasis and regulation of insulin sensitivity [9,10]. Primary bile acids, including cholic acid (CA), chenodeoxycholic acid (CDCA) and hyocholic acid (HCA), are synthesized by hepatocytes. Following conjugation in the liver with glycine or taurine, CA forms glycocholic acid (GCA) or taurocholic acid (TCA), whereas CDCA forms glycochenodeoxycholic acid (GCDCA) or taurochenodeoxycholic acid (TCDCA). These conjugated primary bile acids are secreted into the bile and released into the intestinal lumen, where they are unconjugated by bacterial metabolism, and facilitate emulsification and absorption of lipids and fat-soluble vitamins [11,12]. In the intestine, gut anaerobes of the genera *Bacteroides* and *Clostridium* convert CA to deoxycholic acid (DCA) and CDCA to lithocholic acid (LCA), hyodeoxycholic acid (HDCA) and ursodeoxycholic acid (UDCA) [13,14]. Under normal conditions, over 95% of conjugated and unconjugated secondary bile acids are reabsorbed and recycled via the portal vein into the liver [15,16]. At high concentrations, secondary bile acids cause cell membrane damage, resulting in focal destruction of intestinal epithelium. This induces an inflammatory reaction and hyperproliferation of undifferentiated cells, potentiating transition into a precancerous state [17]. The role of bile acids and type and species of intestinal bacteria involved in the transformation of bile acids were extensively reviewed in a recent publication by Jia et al. [16] 

Patients with CRC have been shown to have elevated levels of secondary bile acids in fecal samples [18,19,20], suggesting a potential role for these metabolites in carcinogenesis. DCA at physiological concentrations activates signaling pathways that lead to selective resistance to apoptosis, angiogenesis, proliferation and oxidative stress [21,22]. Dietary DCA also induces development of colon adenomas or cancer in mouse models [23]. Prior human studies have used fecal samples to report high concentration of LCA [24], and DCA [25,26], and total secondary bile acids in CRC cases versus controls [24]. Healthy rural Africans had significantly lower concentrations of primary and secondary bile acids compared to healthy African-Americans, who have a higher risk of CRC [27]. Two other studies have examined the associations between bile acids and CRC. Accordingly, a study by Kühn et al. [28], using plasma samples, found a positive association between glycohyocholic acid (GHCA) and CRC risk, whereas Weir et al. [25] examined fecal samples and reported that UDCA level was elevated in healthy controls compared to that of colon cancer patients. While blood or fecal samples are logistically easier to acquire, they are not necessarily representative of gut bacterial composition [26,29] or gut bile acid profiles. There is also a gap to be filled in understanding the role of bile acids in colorectal adenomas, particularly advanced adenoma, an important precursor of CRC, because these prior studies only evaluated the roles of bile acids in CRC risk.

The objective of this study was to characterize changes in the ileal bile acid profiles in a case-control study design with 14 advanced adenomas and 32 participants who were free of cancer or any adenomas. 

## 2. Methods

### 2.1. Study Population

The current analysis was a proof-of-concept study with a case-control design, comprising 46 participants (i.e., 14 advanced adenomas, defined as any adenoma with maximum diameter ≥ 1 cm, high-grade dysplasia, or with tubulovillous or villous histology [6], and 32 free of adenoma and CRC) recruited at Vinmec Healthcare System in Hanoi, Vietnam. All cases were males and females aged 40–75 years old with a new pathologic diagnosis of advanced adenoma. Controls were matched to cases by frequency of age (5-year group) and sex. A short questionnaire was used in the in-person interview, which was conducted by a trained interviewer to obtain information of demographics, history of cigarette smoking, alcohol intake, medical history and medication use. Adenoma status was confirmed by pathologic evaluation. 

Study participants provided the following: (1) ileal juice (2 mL) obtained by inserting the colonoscope into the terminal ileum and aspirating into a trap or an extractor (DVR-3858-Symphon Co., Ltd., Tongluo Township, Miaoli County, Taiwan, R.O.C); ileal fluid was transferred into a 2 mL tube containing 1 mL RNA*later* RNA Stabilization Reagent following standardized protocols [30]; (2) fecal samples collected prior to colonoscopy, using Zymo Research kit (DNA/RNA) Shield Fecal Collection Tube, following protocol of UPMC Center for Medicine & Microbiome (http://www.microbiome.pitt.edu/wp-content/uploads/2017/08/Stool-Instructions-to-Participants-FINAL.pdf (accessed on 25 May, 2023)); (3) a 10 mL fasting blood (EDTA acid vacutainer); and (4) a mouth rinse sample. All collected samples were immediately placed in insulated boxes with ice (0–2 °C) after the collection and were processed within 4 h for long-term storage at −80 °C. REDCap [31] was used to store and track (real-time) both questionnaire data and sample inventory for each study participant. 

All study participants provided written consents before participating in the study. The study was approved by the Institutional Review Boards (IRBs) of the Vinmec Healthcare System and the University of Pittsburgh. 

### 2.2. Metabolomic Assay

Ileal bile acids were quantified by ultraperformance liquid chromatography triple-quadrupole mass spectrometry (LC-TQMS) assays as described previously [32,33,34]. Briefly, each 100 µL of ileal juice was lyophilized to dry powder in a BA-free matrix using a freeze dryer. The residues were reconstituted in 1:1 (*v*/*v*) mobile phase B (acetonitrile/methanol = 95:5, *v*/*v*) and mobile phase A (water with formic acid) and centrifuged at 13,500× *g* and 4 °C for 20 min. The supernatant was then transferred to a 96-well plate for LC-TQMS analysis. A UPLC-TQMS) system (ACQUITY UPLC-Xevo TQ-S, Waters Corp., Milford, MA, USA) was used to quantify BAs in the human samples. The raw data were processed using the TargetLynx application manager (Waters Corp., Milford, MA, USA) to obtain calibration equations and the measured concentration of each BA in the individual samples. The intra- and inter-batch CVs are less than 10% and the recovery rate is higher than 95–110% for all BAs in quality control samples in the current study. To ensure the comparability between groups, lab personnel were blinded to disease status of biospecimens. 

### 2.3. Statistical Analysis

Means and standard deviation (SD) were calculated for continuous variables, while counts and proportions were computed for categorical variables. Analysis of variance (ANOVA) and *χ*^2^ test were used to compare the distributions of continuous and categorical variables, respectively, among cases and controls. 

The distributions of ileal bile acids measured were markedly skewed toward high values, which were normalized to a large extent by transformation to natural logarithmic values. Therefore, formal statistical testing was performed on logarithmically transformed values, and geometric means are presented. The analysis of covariance (ANCOVA) was used to examine the difference in absolute and relative levels of BAs between case and control status with adjustment for age, sex, body mass index, smoking status and type 2 diabetes.

We followed the proposal of bile acid grouping by Hofmann et al. (1992) [33]. For the present analysis, the classifications and groups of specified bile acids are depicted in Supplementary Table S1. We also calculated the ratios of specific secondary bile acids to their parent bile acids, specifically the DCA/CA, LCA/CDCA, UDCA/CDCA species and secondary Bas/primary Bas ratios. Statistical analyses were performed using SAS version 9.4 (SAS Institute Inc., Cary, NC, USA) and R version 3.6. All *p* values were two-sided, and 0.05 was used as a threshold of statistical significance.

## 3. Results

Cases were more likely to be male and current drinkers (both *p*-values < 0.05). There was no difference between cases and controls for age, BMI, smoking status, history of diabetes and medication use (i.e., antibiotics in the past 6 months, metformin, statin, aspirin or other NSAIDs) (Table 1).

**Figure 1:**
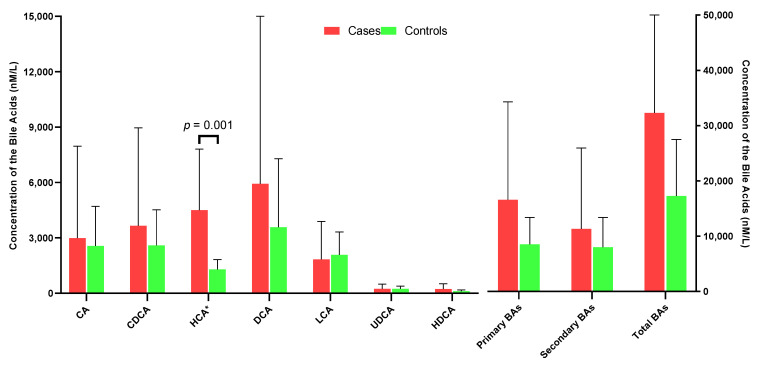
Mean concentrations of ileum CA, CDCA, CDA, LCA, UDCA and primary BAs, secondary BAs and total BAs in case and control groups.

**Figure 2:**
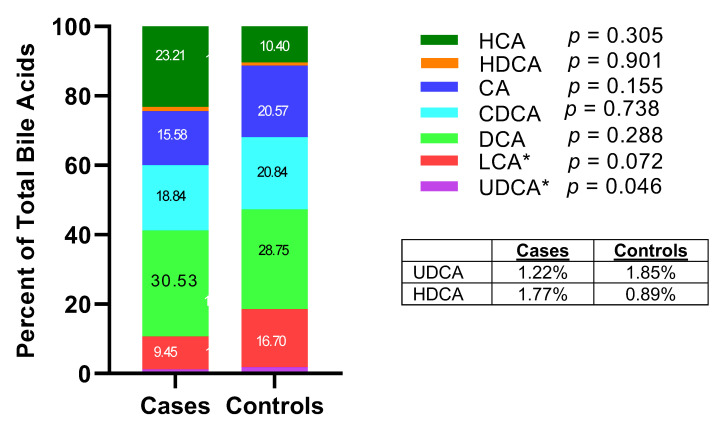
Percentage of specific bile acid species in total bile acids in case and control groups (* *p* < 0.05).

Compared with controls, cases had a non-statistically significant higher level of summed total bile acids (*p* = 0.196), summed primary (*p* = 0.158) and summed secondary bile acids (*p* = 0.514), respectively (Figure 1, Supplementary Table S2). Among the primary bile acids, HCA species was the most abundant, followed by CDCA species and CA species. Cases had significantly higher concentrations of HCA species than controls (geometric mean = 4501.0 versus 1292.3 nM, *p* = 0.001). When measured as percentage of total bile acids, the relative abundance of UDCA in cases was significantly lower than that in controls (1.22% versus 1.85%, *p* = 0.046) (Figure 2).

**Figure 3:**
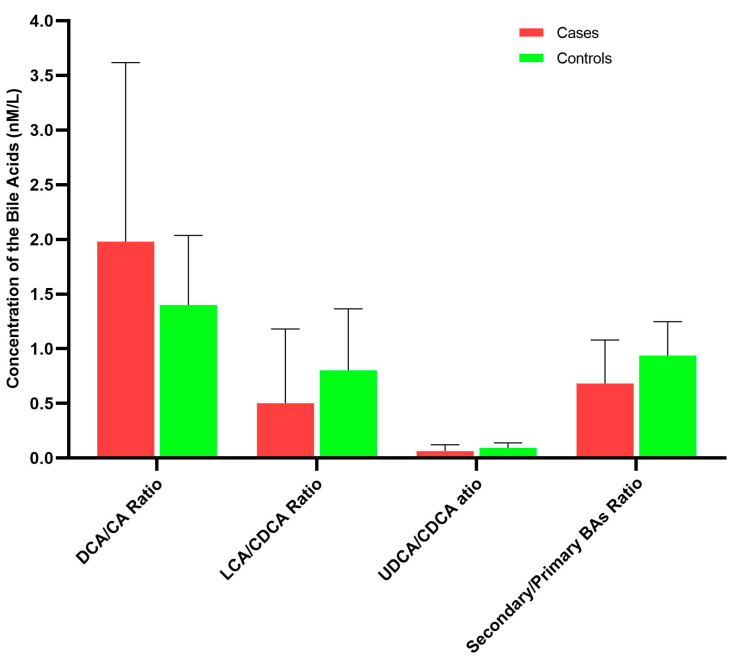
Ratios of selected bile acid species in case and control groups.

The ratios of secondary bile acids to primary bile acids reflected the abundance and functional activities of gut microbiota that produced secondary bile acids from their corresponding primary bile acids. Compared with controls, cases had lower ratio of total secondary to total primary bile acids, although this difference was not statistically significant (0.68 versus 0.94, *p* = 0.286; Figure 3, Supplementary Table S2). For the ratios of individual bile acid species, the ratios of UDCA species to CDCA species and LCA species to CDCA species were lower, whereas the ratio of DCA species to CDCA species was higher in cases than in controls, but these differences were not statistically significant.

Both DCA and LCA species were positively correlated with metformin, while HCA and total primary BAs were negatively correlated with statin (all *p*s < 0.05) (Supplementary Table S3). No significant correlation was observed for bile acid species with patient characteristics, cigarette smoking, alcohol intake, history of diabetes, and use of aspirin or other non-steroidal anti-inflammatory drugs (NSAIDs) (Supplementary Table S3). 

**Figure 4:**
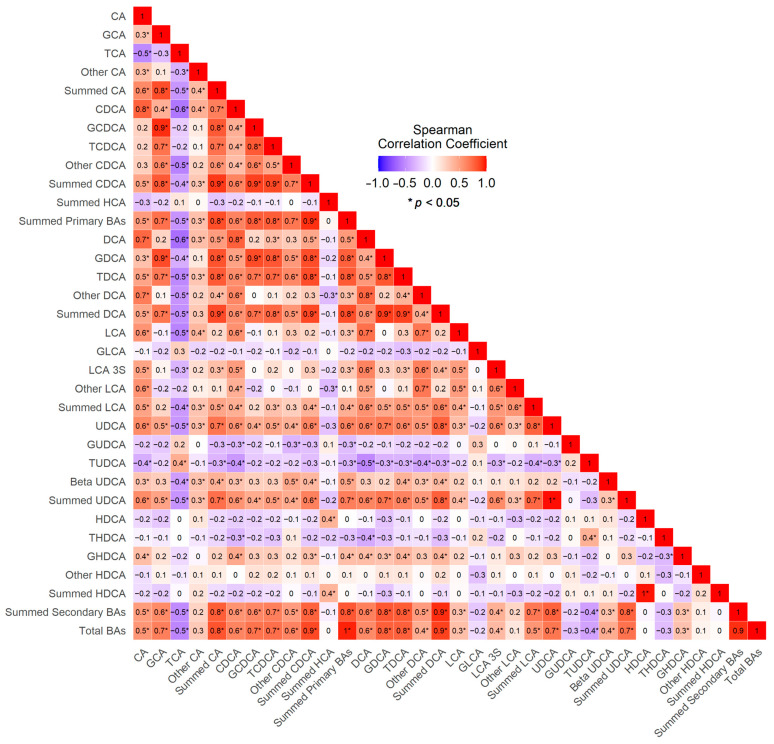
Correlogram of the Spearman’s Correlations between Bile Acids.

Overall, the concentrations of summed secondary bile acid species in ileal juice were strongly correlated with the summed primary bile acid species (Spearman Correlation Coefficient (*r*) = 0.93, *p* < 0.01) (Figure 4). Among specific bile acid species, the summed CA species in ileal juice was highly correlated with the summed CDCA species and the summed DCA species (*r* ≥ 0.80, both *p*s < 0.01), whereas the summed UDCA species was moderately correlated with the summed CA species (*r* = 0.717), the summed CDCA species (*r* = 0.643), the summed DCA species (*r* = 0.779) and the summed LCA species (*r* = 0.747) (all *p*s < 0.05). Interestingly, TCA and TUDCA were inversely correlated with most of the bile acids measured. 

## 4. Discussion

The current study, which collected ileal juice from patients with biopsy-confirmed colorectal advanced adenomas, provides deep understanding of the complex inter-relationship between colorectal advanced adenoma pathogenesis and bile acid physiology. We found that the concentrations of HCA species in ileum juice were significantly elevated in patients with advanced adenoma. In addition, the relative abundance of UDCA was significantly lower in patients with advanced adenoma than in those free of adenoma and cancer. The present study did not show significant differences in ileum juice concentrations of total and individual primary bile acids including CA and CDCA as well as total and individual secondary bile acids including DCA, HDCA and LCA.

The primary bile acids, CA and CDCA, are produced in the liver via two complex multistep biosynthetic pathways and then conjugated by either taurine and/or glycine to enhance their water solubility. Bile acids first flow into bile and then are released into the small intestine after a meal where they facilitate digestion and absorption of fat and fat-soluble vitamins. Secondary bile acids are formed in the intestines by deconjugation and dehydroxylation of primary bile acids, and the majority of bile acids (95%) are reabsorbed in the brush border membrane of the terminal ileum and undergo enterohepatic circulation [15,16]. 

We observed a higher concentration level of ileum juice HCA in cases than in control subjects. It is noted that two main primary bile acids, CA and CDCA, are present in most mammals [35], and there are species-specific differences in bile acid synthesis, transport and metabolism, particularly the composition of the bile acid pool. Accordingly, CDCA might be partly 6α-hydroxylated into αHCA by CYP3A [36], while murine animals might have additional primary BA, such as muricholic acids (e.g., αMCA and βMCA) due to the presence of 6β-hydroxylase [37,38]. Recently, 6β-hydroxylase has been identified as CYP2C22 in rats and Cyp2c70 in mice, both of which are similar to CYP2C9 in humans, which has the function of a drug-metabolizing enzyme without BA oxidation activities [39]. 

In a nested case-control study of 569 colon cancer cases matched with 569 controls within the European Prospective Investigation into Cancer and Nutrition (EPIC) cohort, Kűhn et al. [26] recently reported that higher level of serum concentration of glycohyocholic acid (GHCA) was associated with increased risk of colon cancer (OR = 1.65, 95% CI: 1.13–2.40). While this is suggestive evidence on the role of HCA in colorectal cancer, its role in colorectal adenomas and progression from adenomas to cancer is much less understood and thus warrants further research. 

In the current study, we found that the relative abundance of UDCA species was significantly lower in cases than in controls. This finding is consistent with results from a prior study by Weir et al. [27] that evaluated fecal samples of 10 healthy individuals and 11 patients prior to surgical resection for colon cancer and found increased levels of UDCA (approximately 63%) in healthy individuals compared to colon cancer patients. In a large population-based study using data from the Taiwan National Health Insurance Research Database during 2006–2010 period (n = 1,961,788), Huang et al. [40] found that UDCA, a chemo-preventive drug, was associated with reduced risk of colorectal cancer. 

UDCA is considered an important secondary bile acid [41] due to its “protective properties” or metabolic effects ranging from decreasing endoplasmic reticulum stress, anti-apoptotic effects and lowering ileum TNF-α concentrations to improving hepatic insulin sensitivity [42]. It is also noted that UDCA is the 7β-OH epimer of CDCA, a primary bile acid, and the epimerization (or conversion) has two subsequent steps: (1) oxidation of the 7α-hydroxyl group by 7α-acid hydroxysteroid dehydrogenases (or 7α-HSDH) and (2) stereospecific reduction of the 7-keto functionality by 7β-acid hydroxysteroid dehydrogenases (or 7β-HSDH), resulting in the formation of the corresponding 7β-hydroxyl group. Different bacteria such as *E. coli* HB 101 [43], *Clostridium sordelii* [44], *Clostridium scindens* (formerly *Eubacterium* sp VPI 12708) [45] and *Bacteroides fragilis* [46] are found to encode for 7α-HSDH, while *Clostridium absonum* [41,47], *Clostridium limosum* [48] and *Stenotrophomonas* (formerly *Pseudomonas* and *Xanthomonas*) maltophilia [49] are reported to encode for 7β-HSDH. Recently, Wei et al. [50] showed that *Clostridium scidens* is an important bacterial species that can mediate the epimerization process of CDCA to UDCA. Taken together, it is suggested that the lower UDCA in the bile of patients with adenoma reflects lower abundance of these bacteria. Further studies are warranted to elucidate pathways and/or bacteria on the role of UDCA in the progression from advanced adenomas to colorectal cancer.

In clinical settings, UDCA is used in treatment of different outcomes such as primary biliary cholangitis [51] and dissolution of cholesterol gallstones [52,53]. In addition, in a phase III randomized trial of 1285 individuals who had undergone colorectal adenomas removal within the past 6 months and received daily treatment with UDCA (6–10 mg/kg of body weight; n = 661 individuals) or with placebo (n = 624 individuals) for 3 years or until follow-up colonoscopy, Alberts et al. [54] reported that overall, the oral UDCA treatment was associated with non-statistically significant reduction in total colorectal adenoma recurrence but with a statistically significant 39% reduction in recurrence of colorectal adenomas in patients with high-grade dysplasia. Furthermore, in another study using fecal samples in a phase III randomized clinical trial of UDCA for the prevention of colorectal adenomas, Pearson et al. [55] compared the changes in the microbiome composition after a 3-year intervention in a subset of randomized participants including an intervention arm with oral UDCA (8–10 mg/kg body weight/day) (n = 198) and a placebo arm (n = 203). They found that in the intervention arm, the relative abundance of Bacteroidetes was significantly increased, while the relative abundance of Firmicutes decreased significantly in post-intervention subjects compared with subjects at baseline. Additionally, there was no significant change in these genera among subjects in the placebo arm. Both Bacteroidetes and Firmicutes are important microbial phyla of the gut microbiome, and their ratio reflects the dietary pattern and overall balance of the gut microbiome. Accordingly, a high ratio of Firmicutes to Bacteroidetes is found to be associated with Western diet consumption, which is also associated directly to the treatment with UDCA (above-mentioned) [55] and to adverse metabolic changes in obesity patients [56], while its low ratio is associated with reduced gut biodiversity [57] and inflammatory bowel disease (IBD) [58]. 

Our study has several strengths. All study subjects enrolled in our study were well phenotyped with histologic confirmation. Ileum juice samples were collected in both cases and controls at the initial performance of colonoscopy and were transferred to the centralized laboratory for storage for downstream analysis within less than an hour. We used a comprehensive, state-of-the-art LC-TQMS technology that simultaneously quantified absolute concentrations of 42 individual bile acids in ileum juice. The use of ileum juice bile acids had several advantages compared to blood-based bile acids. First, the terminal ileum location is immediately proximal to the location where adenomas and cancer occur. A recent study by Gevers et al. [59] showed that microbiome profiles obtained from ileal biopsies have higher power to predict disease progression in Crohn’s disease patients compared to rectal biopsies or fecal samples, suggesting that ileal juice may be more informative than other sample types commonly used in assessing the role of the microbiome in colorectal lesions. Third, ileal juice avoids the potential problems inherent in the measurement of serum bile acids, including absorption, hepatic metabolism and transportation (such as impaired liver function and gallbladder diseases, etc.). 

Our study also has several limitations. Patients might have taken medication, such as antibiotics, that impacted bile acid pools. However, there was no significant difference in bile acids measured between patients who reported use of antibiotics and those who did not. In addition, patients enrolled in this study were Vietnamese who had different dietary habits; therefore, our findings may not be directly generalizable to other populations. However, given that ileal juice samples were collected after 12–14 h of fasting, the impact of dietary intake on bile acids in ileum juice would be minimal. The other limitation was the cross-sectional study design, which did not allow us to examine temporal effects. In other words, it was unknown whether the differences observed between cases and controls were due to the presence of colorectal advanced adenomas. Despite these limitations, our study provided invaluable insights into the relationship between bile acids in ileal juice and the presence of colorectal adenomas that warrant further study. 

## 5. Conclusions

In conclusion, we found that the concentration of HCA species in ileal juice was significantly elevated in patients with biopsy-confirmed colorectal advanced adenomas compared with normal colonoscopy controls. In contrast, the relative abundance of UDCA in ileal juice was significantly reduced in patients with colorectal adenomas compared with controls. Our results support the growing body of evidence on the important role of the gut microbiome (i.e., *Clostridium sordelii*, *Clostridium scindens*, *Bacteroides fragilis*, *Clostridium absonum, Clostridium limosum* and *Stenotrophomonas*) that convert CDCA into UDCA in the pathogenesis of colorectal adenomas and cancer. Further studies are warranted to replicate our findings and to determine the roles of bile-acid-related bacteria in the development from colorectal adenomas to colorectal cancer. Results from such studies are of significance to early detection and control of CRC.

## Figures and Tables

**Table 1:** Selected Characteristics of Study Participants by Disease Status.

	Cases (n, %)	Controls (n, %)	*p*-Value
No. of subjects	14	32	
Age (Mean ± SD)	62.79 ± 8.05	61.06 ± 10.53	0.71
Gender			
Male	12 (85.7)	12 (37.5)	**0.003**
Female	2 (14.3)	20 (62.5)	
BMI (Mean ± SD)	22.3 ± 2.8	22.6 ± 2.7	0.71
Smoking status			
Never	7 (50.0)	23 (71.9)	0.31
Former smoker	3 (21.4)	5 (15.6)	
Current smoker	4 (20.6)	4 (12.5)	
Current alcohol use ^a^			
No	4 (28.6)	24 (75.0)	**0.007**
Yes	10 (71.4)	8 (25.0)	
History of diabetes			
Yes	12 (85.7)	26 (81.2)	0.71
No	2 (14.3)	6 (18.7)	
Medication usage			
Antibiotic, past 6 m	6 (42.9)	13 (40.6)	0.89
Metformin	1 (7.1)	3 (9.4)	0.80
Statin	3 (21.4)	0 (0.0)	0.79
Aspirin	1 (7.1)	1 (3.1)	0.53
Other NSAIDs	0 (0.0)	1 (3.1)	0.50

^a^ During the past 6 months. Bold: *p* < 0.05.

## Data Availability

Data are available on request. The data underlying this article will be shared at reasonable request to the corresponding author.

## Supplementary Materials

The following supporting information can be downloaded at: https://www.mdpi.com/article/10.3390/nu15132930/s1, Table S1. Classification of Measured Bile Acids. Table S2. Ileum Bile Acids in Cases and Controls in Study Population. Table S3. Correlation Coefficients between Bile acids and Patient’s Characteristics and Clinical Measurements.
