# Supplementary material for: Associations between Ileal Juice Bile Acids and Colorectal Advanced Adenoma
Source: Nutrients. 2023 Jun 28;15(13):2930. doi: 10.3390/nu15132930 (PMC10346326; doi:10.3390/nu15132930)
Supplement: Supplementary file 1 [file nutrients-15-02930-s001.zip › nutrients-2430501-supplementary.pdf]

Supplementary Table S1: Classification of Measured Bile Acids.

Bile acid	Primary/Secondary	Species	Conjugation	Full name
CA	Primary	CA	Unconjugated	Cholic acid
GCA	Primary	CA	Conjugated	Glycocholic acid
TCA	Primary	CA	Conjugated	Taurocholic acid
NorCA	Primary	CA	Conjugated	Norcholic acid
βCA	Primary	CA	Conjugated	β cholic acid
βUCA	Primary	CA	Conjugated	β-ursocholic acid
apoCA	Primary	CA	-	apocholic acid
UCA	Primary	CA	Conjugated	ursocholic acid
CDCA	Primary	CDCA	Unconjugated	Chenodeoxycholic acid
GCDCA	Primary	CDCA	Conjugated	Glycochenodeoxycholic acid
TCDCA	Primary	CDCA	Conjugated	Taurochenodeoxycholic acid
CDCA-24G	Primary	CDCA	Conjugated	Chenodeoxycholic acid 24-glucuronide
7-ketoLCA	Primary	CDCA	Conjugated	7-ketolithocholic acid
GDHCA	Primary	CDCA	Conjugated	Glycodeoxyhyocholic acid
TDHCA	Primary	CDCA	Conjugated	Taurodehydrocholic acid
HCA	Primary	HCA	Unconjugated	Hyocholic acid
GHCA	Primary	HCA	Conjugated	Glycohydrocholic acid
THCA	Primary	HCA	Conjugated	Taurohydrocholic acid
12-DHCA	Primary	HCA	Conjugated	12-Dehydrocholic acid
TωMCA	Primary	HCA	Conjugated	Tauro ω-murichollic acid
TαMCA	Primary	HCA	Conjugated	tauro α-muricholic acid
DCA	Secondary	DCA	Unconjugated	Deoxy-cholic acid
GDCA	Secondary	DCA	Conjugated	Glycodeoxycholic acid
TDCA	Secondary	DCA	Conjugated	Taurodeoxycholic acid
isoDCA	Secondary	DCA	Conjugated	Isodeoxycholic acid
NorDCA	Secondary	DCA	Conjugated	Nordeoxycholic acid
12-ketoLCA	Secondary	DCA	Conjugated	12-ketolithocholic acid
7-ketoDCA	Secondary	DCA	Conjugated	7-Ketodeoxycholic Acid
LCA	Secondary	LCA	Unconjugated	Lithocholic acid
GLCA	Secondary	LCA	Conjugated	Glycolithocholic acid
6,7-dikotoLCA	Secondary	LCA	Conjugated	6,7-dikotolithocholic acid
isoLCA	Secondary	LCA	Conjugated	Isolithocholic acid
alloLCA	Secondary	LCA	Conjugated	Allolithocholic acid
LCA-3S	Secondary	LCA	Conjugated	Lithocholic acid-3-sulfate
GLCA-3S	Secondary	LCA	Conjugated	Glycolithocholic acid 3 sulfate
7,12-diketoLCA	Secondary	LCA	Conjugated	7,12-diketolithocholic acid
dehydroLCA	Secondary	LCA	Conjugated	dehydrolithocholic acid
UDCA	Secondary	UDCA	Unconjugated	Ursodeoxycholic acid
GUDCA	Secondary	UDCA	Conjugated	Glycoursodeoxycholic acid
TUDCA	Secondary	UDCA	Conjugated	Tauroursodeoxycholic acid
βUDCA	Secondary	UDCA	Conjugated	β-ursodeoxycholic acid
HDCA	Secondary	HDCA	Unconjugated	Hyodeoxycholic acid
GHDCA	Secondary	HDCA	Conjugated	Glycol-hyodeoxycholic acid
THDCA	Secondary	HDCA	Conjugated	Tauro-hyodeoxycholic acid
βHDCA	Secondary	HDCA	Conjugated	β-hyodeoxycholic acid
6-ketoLCA	Secondary	HDCA	Conjugated	6-ketolithocholic acid
ωMCA	Secondary	HDCA	Conjugated	ω muricholic acid

Supplementary Table S2. Ileum Bile Acids in Cases and Controls in Study Population.

	Geometric means (nM)*		
	**Cases (n=14)**	**Controls (n=32)**	***P-value***
**Primary BAs**			
CA Species			
CA (nM)	321.3	214.2	0.667
GCA (nM)	2,081.5	766.8	0.246
TCA (nM)	1.46	2.14	0.588
Other CAs (NorCA + βCA+ UCA + 7_ketolCA) (nM)	158.2	173.2	0.795
Summed CA species (nM)	2,989.6	2,555.7	0.803
CDCA Species			
CDCA (nM)	647.0	341.0	0.352
GCDCA (nM)	1,388.5	325.5	0.571
TCDCA (nM)	287.1	160.2	0.482
Other CDCAs (CDCA-24G+TDHCA) (nM)	371.8	375.0	0.985
Summed CDCA species (nM)	3,655.4	2,590.0	0.550
HCA Species			
GHCA (nM)	2.84	4.12	0.374
THCA (nM)	17.3	11.8	0.434
12-DHCA (nM)	5.24	4.94	0.911
TωMCA (nM)	6.46	5.72	0.796
HCA (nM)	**4,412.8**	**1,216.1**	**0.001**
Summed HCA species (nM)	**4.501.9**	**1,292.3**	**0.001**
Total primary BAs (nM)	16,599.0	8,527.3	0.158
**Secondary BAs**			
DCA Species			
DCA (nM)	250.3	175.9	0.629
GDCA (nM)	4,060.4	2,232.2	0.432
TDCA (nM)	289.0	146.5	0.455
Other DCAs (isoDCA+ NorDCA+7-ketoDCA) (nM)	85.5	79.7	0.784
Summed DCA species (nM)	5,920.9	3,574.6	0.493
LCA Species			
LCA (nM)	58.3	81.4	0.712
GLCA (nM)	0.24	0.24	0.934
GLCA-3S (nM)	251.6	226.3	0.835
Other LCAs (isoLCA+alloLCA+LCA-3S+ Dehydroxy-LCA+6,7-diketoLCA+ 7,12_diketoLCA) (nM)	689.8	882.1	0.534
Summed LCA species (nM)	1,835.6	2,075.9	0.799
UDCA Species			
UDCA (nM)	154.1	158.4	0.965
GUDCA (nM)	2.60	2.62	0.632
TUDCA (nM)	2.16	2.80	0.627
βUDCA (nM)	27.84	21.22	0.436
Summed UDCA species (nM)	237.1	240.5	0.976
HDCA Species			

HDCA (nM)	132.5	64.5	0.308
THDCA (nM)	4.6	6.6	0.439
GHDCA (nM)	3.6	2.5	0.208
Other HDCAs (βHDCA + ωMCA) (nM)	11.1	11.3	0.965
Summed HDCA species (nM)	227.4	111.1	0.173
Total Secondary BAs (nM)	11,320.0	7,986.3	0.514
**Total BAs (primary + secondary BA species)**	32,288	17,257	0.196
**Ratios of BAs Species**			
DCA/CA Spec ratio	1.98	1.40	0.371
LCA/CDCA species ratio	0.50	0.80	0.397
LCA/(CDCA+UDCA) species	0.46	0.70	0.420
UDCA/CDCA species ratio	0.06	0.09	0.388
Sec BAs/Pri BAs ratio	0.68	0.94	0.286

* Adjusted for age, sex, body mass index, smoking status, drinking status, and type 2 diabetes

Abbreviations: See details in Supplementary Table 1- Classification of Measured Bile Acids

Supplementary Table S3. Correlation Coefficients between Bile acids and Patient's Characteristics and Clinical Measurements.

	CA Species	CDCA Species	HCA Species	Total Primary BAs	DCA Species	LCA Species	UDCA Species	HDCA Species	Total Secondary BAs	Total BAs
Age	0.110	0.102	-0.230	0.099	0.029	0.069	-0.037	0.020	0.088	0.120
Sex	0-.134	-0.226	-0.043	-0.180	0.010	-0.039	-0.056	0.025	-0.052	-0.144
BMI	0.281	0.114	-0.019	0.179	0.238	0.159	0.159	0.217	0.265	0.230
Smoking status	-0.023	0.050	0.049	0.046	-0.154	-0.200	-0.132	0-.045	-0.103	0.019
Drinking status	-0.171	-0.225	-0.231	-0.252	-0.094	-.050	-0.033	-0.022	-0.154	-0.258
History of diabetes	0.026	0.022	-0.272	0.009	0.091	0.138	0.060	0.147	0.168	0.104
Antibiotic, past 6m	-0.061	-0.022	0.105	0.042	0.028	0.025	-0.075	0.030	0.098	0.085
Metformin	0.250	0.273	-0.267	0.238	**0.325***	**0.360***	0.261	0.087	0.314	0.279
Statin	-0.106	-0.225	**-0.313***	**-0.290***	-0.136	0.013	-0.106	-0.125	-0.129	-0.248
Aspirin	0.153	0.193	-0.185	0.128	0.225	0.217	0.193	-0.064	0.201	0.161
Other NSAIDs	-0.219	-0.253	0.028	-0.219	-0.208	0.028	-0.241	-0.219	-0.163	-0.185

*P < .05

Abbreviations: See details in Supplementary Table 1- Classification of Measured Bile Acids
